# Risk factors for adverse outcomes in women with high-risk pregnancy and their neonates, Haiti

**DOI:** 10.26633/RPSP.2021.147

**Published:** 2021-11-01

**Authors:** Jorien Schuurmans, Emily Borgundvaag, Pasquale Finaldi, Rodnie Senat-Delva, Fedner Desauguste, Colette Badjo, Michiel Lekkerkerker, Reynaldo Grandpierre, Gerald Lerebours, Cono Ariti, Annick Lenglet

**Affiliations:** 1 Médecins Sans Frontières Port-au-Prince Haiti Médecins Sans Frontières, Port-au-Prince, Haiti; 2 Médecins Sans Frontières Amsterdam Netherlands Médecins Sans Frontières, Amsterdam, The Netherlands; 3 Ministère de la Santé Publique et de la Population Port-au-Prince Haiti Ministère de la Santé Publique et de la Population, Port-au-Prince, Haiti; 4 National Bioethics Committee Port-au-Prince Haiti National Bioethics Committee, Port-au-Prince, Haiti; 5 Centre for Trials Research Cardiff University School of Medicine Cardiff United Kingdom Centre for Trials Research, Cardiff University School of Medicine, Cardiff, United Kingdom

**Keywords:** Maternal death, stillbirth, birth weight, pregnancy complications, pre-eclampsia, Haiti, Muerte materna, mortinato, peso al nacer, complicaciones del embarazo, pre-eclampsia, Haití, Morte materna, natimorto, peso ao nascer, complicações na gravidez, pre-eclampsia, Haiti

## Abstract

**Objectives.:**

To determine the prevalence of maternal death, stillbirth and low birthweight in women with (pre-)eclampsia and complicated pregnancies or deliveries in Centre de Références des Urgences Obstétricales, an obstetric emergency hospital in Port-au-Prince, Haiti, and to identify the main risk factors for these adverse pregnancy outcomes.

**Methods.:**

We conducted a retrospective cohort study of pregnant women admitted to Centre de Référence des Urgences Obstétricales between 2013 and 2018 using hospital records. Risk factors investigated were age group, type of pregnancy (singleton, multiple), type of delivery and use of antenatal care services.

**Results.:**

A total of 31 509 women and 24 983 deliveries were included in the analysis. Among these, 204 (0.6%) maternal deaths (648 per 100 000 women giving birth), 1962 (7.9%) stillbirths and 11 008 (44.1%) low birthweight neonates were identified. Of all admissions, 10 991 (34.9%) were women with (pre-)eclampsia. Caesarean section significantly increased the risk of maternal death in the women with a complicated pregnancy and women with (pre-)eclampsia, but reduced the risk of stillbirth in such women. Not attending antenatal care was associated with a significantly higher risk of stillbirth (odds ratio (OR) 4.82; 95% confidence interval (CI) 3.55–6.55) and low birthweight (OR 1.40; 95% CI 1.05–1.86) for women with complicated pregnancies.

**Conclusion.:**

To prevent and treat pregnancy complications as early as possible, antenatal care attendance is crucial. Improving the quality of and access to antenatal care services and providing it free to all pregnant women in Haiti is recommended.

## RESUMEN

**Objetivos.:**

Determinar la prevalencia de la muerte materna, la mortinatalidad y el peso bajo al nacer en mujeres con eclampsia o preeclampsia y embarazos o partos complicados en el Centre de Références des Urgences Obstétricales, un hospital de urgencia obstétrica en Puerto Príncipe (Haití), e identificar los principales factores de riesgo de estos resultados adversos del embarazo.

**Métodos.:**

Se llevó a cabo un estudio de cohortes retrospectivo con embarazadas ingresadas en el Centre de Références des Urgences Obstétricales entre el año 2013 y el 2018 empleando los registros del hospital. Se investigaron como factores de riesgo el grupo etario, el tipo de embarazo (único o múltiple), el tipo de parto y el uso de los servicios de atención prenatal.

**Resultados.:**

Se incluyó en el análisis un total de 31 509 mujeres y 24 983 partos. De estos, se documentaron 204 (0,6 %) muertes maternas (648 de cada 100 000 mujeres que dan a luz), 1962 (7,9 %) mortinatos y 11 008 (44,1%) recién nacidos con peso bajo al nacer. De todos los ingresos, 10 991 (34,9 %) fueron mujeres con eclampsia y preeclampsia. La cesárea aumentó significativamente el riesgo de muerte materna en mujeres con un embarazo complicado y mujeres con eclampsia y preeclampsia, si bien redujo el riesgo de mortinatalidad en estas mujeres. No asistir a la atención prenatal se asoció con un riesgo significativamente mayor de mortinatalidad (razón de posibilidades [OR] 4,82; intervalo de confianza [IC] de 95 % 3,55-6,55) y peso bajo al nacer (OR 1,40; IC de 95 % 1,05-1,86) en mujeres con embarazos complicados.

**Conclusiones.:**

Para prevenir y tratar las complicaciones del embarazo lo antes posible, es crucial asistir a la atención prenatal. Se recomienda mejorar el acceso a servicios de atención prenatal de buena calidad y ofrecerlos de manera gratuita a todas las embarazadas en Haití.

## RESUMO

**Objetivos.:**

Determinar a prevalência de mortalidade materna, natimortos e baixo peso ao nascer em mulheres em pré-eclâmpsia, ou gravidez ou partos com complicações no *Centre de Références des Urgences Obstétricales*, um hospital de emergência obstétrica em Porto Príncipe, no Haiti, e identificar os principais fatores de risco para esses desfechos adversos na gravidez.

**Métodos.:**

Realizamos um estudo de coorte retrospectivo de gestantes admitidas no *Centre de Références des Urgences Obstétricales* entre 2013 e 2018, utilizando os prontuários do hospital. Os fatores de risco estudados foram: faixa etária, tipo de gravidez (feto único ou múltiplo), tipo de parto e uso de serviços de atendimento pré-natal.

**Resultados.:**

A análise incluiu um total de 31.509 mulheres e 24.983 partos. Entre eles, 204 (0,6%) mortes maternas (648 por 100.000 mulheres que deram à luz), 1.962 (7,9%) natimortos e 11.008 (44,1%) recém-nascidos com baixo peso ao nascer estavam documentados. De todas as internações, 10.991 (34,9%) eram de mulheres em pré-eclâmpsia. Parto cesariana aumentou significativamente o risco de mortalidade materna nas mulheres com complicações na gravidez e com pré-eclâmpsia, mas reduziu o risco de natimorto nessas mulheres. Não fazer acompanhamento pré-natal foi associado a risco significativamente maior de natimorto (razão de chances (OR) 4,82; intervalo de confiança de 95% (IC) 3,55-6,55) e baixo peso ao nascer (OR 1,40; IC de 95% 1,05-1,86) para as mulheres com complicações na gravidez.

**Conclusões.:**

Para prevenir e tratar as complicações da gravidez o mais precocemente possível, o acompanhamento pré-natal é crucial. Recomenda-se o aprimoramento do acesso a serviços de atendimento pré-natal de qualidade e oferecidos gratuitamente a todas as gestantes do Haiti.

Globally, an estimated 290 000 maternal deaths, 2 million stillbirths and 2.5 million neonatal deaths occur each year (1). Lower and middle-income countries bear the greatest burden of these deaths as 94% of all maternal and child deaths occur in these countries (2). Haiti is the poorest country in the Caribbean, is highly dependent on foreign aid and suffers from the highest maternal and neonatal mortality in the Western hemisphere (3, 4). While maternal and neonatal mortality rates in Haiti have fallen substantially since the 1960s, little progress has been made in the past 2 decades (5, 6). In 2018, the maternal mortality rates in Haiti were 529 women per 100 000 live births and notably exceeded the rate in the Dominican Republic, Haiti’s neighbouring country, which recorded a five-fold lower maternal mortality than Haiti (6).

The main barriers to improving maternal and neonatal health in Haiti are poverty, lack of access to health care, poor transportation, lack of health care facilities and lack of skilled birth attendants. More than 50% of the Haitian population lives below the poverty line of less than US$ 2.41 a day (7). In addition, Haiti is suffering from a free-falling currency, high cost of living and a 13% annual inflation rate in 2017 (7). It is estimated that almost 50% of the Haitian population lacks access to health care because of financial and geographical barriers (4). Infrastructure is poor and the effects of urban congestion contribute to the inability of people to seek health care (8). Other barriers are inadequate health care facilities and the shortage of health workers (4, 9, 10).

Factors associated with maternal death include multiple pregnancy (11), type of delivery (12) and attending antenatal care (13). In general, improving access to antenatal care decreases maternal mortality as well as negative neonatal outcomes (13). Although the association between poor access to antenatal care and maternal mortality is unclear in Haiti, it is known that the antenatal care uptake in Haiti is low with only 67% of pregnant women accessing the recommended four antenatal care visits (14). Furthermore, only 37% of births take place in a health facility attended by a skilled birth attendant, compared to 70% in other low-income countries (15, 16). In Haiti, complications occur in about 40% of pregnancies, while life-threatening complications occur in 15% (17). Nevertheless, most complicated pregnancies have early warning signs and health care has proven to be effective in reducing maternal mortality rates (18). However, pregnant women in Haiti, especially women in the lowest household income, face substantial barriers to accessing antenatal care and skilled birth attendance (17, 19). The main constraints are financial and geographical access, lack of service coverage, lack of transportation and poor quality of care (18, 19).

Stillbirths are often associated with early gestational age, placental abruption, maternal death and complications from prolonged labor. Other risk factors for stillbirth are women not attending antenatal care (20), multiple pregnancy (21) and advanced maternal age (22). Studies show that the number of stillbirths could be reduced by improved (access to) obstetric care during labor and delivery and caesarean sections are often needed to reduce this risk (14, 20).

The preterm birth rate in Haiti was 14.1% in 2010 and the average percentage of neonates with low birthweight (LBW) was 23% (4). Neonates with LBW are 20 times more likely to die than neonates with a normal birthweight (4). Research has shown that mothers younger than 20 years have higher risks of prematurity and LBW (23, 24). Furthermore, multiple pregnancy is associated with LBW. However, these findings have been mainly attributed to the effects of earlier gestational age in twins and triplets than in singletons (25). Neonates with LBW are more likely to be delivered by caesarean section to reduce the risk of neonatal mortality (26).

Between 2011 and 2018, Médecins sans Frontières operated the Centre de Référence pour les Urgences Obstétricales, an obstetric emergency hospital and neonatal care unit in Port-au-Prince, Haiti, which aimed to serve women with high-risk pregnancies. This centre was established in response to the alarming maternal mortality indicators in Port-au-Prince. Even though maternal health is a public health priority in Haiti, there is limited evidence on the factors that lead to adverse pregnancy outcomes in women with a complicated pregnancy or delivery and with (pre-) eclampsia in urban Haiti. Maternal admission data over 5 years provide an opportunity to assess admissions and outcomes and determine the main risk factors for maternal death, stillbirth and LBW. Such data can help guide health care policies to reduce the risk pregnancy outcomes among Haitian women. The objectives of this study therefore were to determine: 1) the prevalence of maternal death, stillbirth and LBW in women with (pre-)eclampsia and complicated pregnancies or deliveries attending the Centre de Référence pour les Urgences Obstétricales; and 2) the main risk factors for adverse pregnancy outcomes in these women.

## MATERIALS AND METHODS

### Study design and setting

This retrospective cohort study was conducted in the Centre de Référence pour les Urgences Obstétricales in Haiti. Through this hospital, Médecins Sans Frontières provided free emergency care to women with high-risk pregnancies and care for their newborn babies. It was the main facility for obstetric and neonatal care in metropolitan Port-au-Prince at the time. The hospital closed in July 2018 for a number of reasons, including the deterioration of what was a temporary structure and the very high costs of turning it into a permanent facility.

### Study participants

All pregnant women and their newborn babies who were admitted to the Centre de Référence pour les Urgences Obstétricales between January 2013 and June 2018 were included in this study. Maternal admissions with suspected cholera were excluded.

### Definitions

Maternal death was defined as the death of a woman while pregnant, irrespective of the duration and site of the pregnancy (ectopic or in the uterus), from any cause related to or aggravated by the pregnancy or its management, but not from accidental or incidental causes. Women who died within 42 days of termination of pregnancy were excluded, as it was not possible to follow up women after discharge from the hospital. Stillbirth was defined as any infant born at gestational age of 22 weeks or more or weighing more than 500 g who did not show signs of life at time of delivery. A LBW neonate was any baby with a birthweight less than 2500 g (27) The following risk factors for negative outcomes were explored in the analysis: age of women admitted (< 20 years, 20–34 years, ≥ 35 years); singleton birth or multiple birth; type of delivery (uncomplicated vaginal delivery, complicated non-instrumental vaginal delivery, instrumental vaginal delivery or caesarean section); and attendance of antenatal care (at least one visit). An uncomplicated vaginal delivery was defined as a term pregnancy, cephalic presentation, singleton birth, and vaginal delivery (including cases with minimal or no assistance, with or without episiotomy). A complicated non-instrumental vaginal delivery was defined as all non-instrumental vaginal deliveries that do not fit the definition of normal spontaneous vaginal delivery (e.g. augmentation of labor, breech delivery, multiple pregnancy, and any instrumental manipulation or assistance). An instrumental vaginal delivery was defined as any delivery assisted by forceps or vacuum extraction. A caesarean section was defined as any delivery made by caesarean section.

The main risk factors for maternal death, stillbirth and LBW were assessed in three pregnancy categories: uncomplicated pregnancy, women with (pre)-eclampsia, and complicated pregnancy. The analysis was stratified by pregnancy category to reduce the risk of confounding. Uncomplicated pregnancies were defined as a singleton pregnancy without maternal morbidities, LBW or delivery complications. A maternal admission with (pre)-eclampsia was defined as a pregnant woman admitted to the Centre de Référence pour les Urgences Obstétricales between 20 weeks of pregnancy and early postpartum who experienced convulsive seizures in the context of pre-eclampsia without other cause. Pre-eclampsia was defined as hypertension (diastolic blood pressure constantly < 90 mmHg), proteinuria (a urine dipstick reading of greater than 2+), and upper body edema that appears suddenly or rapidly worsens. A complicated pregnancy was defined as all complicated pregnancies without eclampsia and pre-eclampsia, with or without delivery complications. This category included pregnancy complications (e.g. antepartum hemorrhage, premature labor, scarred uterus, antepartum infection, hypertension, and fetal distress) and/or delivery complications (e.g. postpartum hemorrhage, obstructed labor, ruptured uterus and other complications).

### Statistical analysis

Maternal admissions were described by frequencies and proportions, stratified by risk factor. The chi-squared test was used to test whether the frequency of negative maternal outcomes differed between pregnancy categories. Univariable associations were calculated using logistic regression models. Stratified analyses were conducted by pregnancy category (complicated pregnancy and (pre)-eclampsia). Women with an uncomplicated pregnancy were excluded from the regression analysis as none of the negative outcomes occurred in this group of women. For the regression model for LBW, the delivery category was excluded because of possible reverse causality.

A multivariable logistic regression analysis was done for risk factors for each pregnancy category (complicated pregnancy/delivery and (pre-)eclampsia) using stepwise backward elimination. To investigate the interaction between type of delivery and pregnancy category, and associations with the other potential risk factors, an interaction term between type of delivery and pregnancy category was introduced into the final regression models. First, the overall interaction was tested and if the interaction effect was < 0.05, more exploratory analysis was conducted. This was done for all outcomes except for LBW.

All data cleaning and analysis were performed with Stata, version 15 (StataCorp. LP, College Station, United States of America).

### Ethical considerations

The study was approved by the National Bioethical Committee of the Ministry of Public Health and Population of Haiti. This research fulfilled the exemption criteria set by the Ethics Review Board of Médecins Sans Frontières for a posteriori analyses of routinely collected clinical data and thus did not require review by the Médecins Sans Frontières Ethics Review Board. The study only used sources of information without personal identifiers.

## RESULTS

A total of 31 509 women were admitted to the Centre de Référence pour les Urgences Obstétricales between January 2013 to June 2018. Eight observations were excluded due to poor data quality; thus, 31 501 maternal admissions were included in the cohort to estimate risk factors for maternal death. In the same period, 24 978 deliveries were recorded. All 24 978 deliveries were included to estimate risk factors for stillbirth, but 40 deliveries were excluded from the analysis on LBW because of missing data on neonatal birthweight; thus 24 938 deliveries were included in the LBW analysis.

Most women admitted experienced a complicated pregnancy (15 655; 49.7%); obstructed labor, antepartum hemorrhage and follow-up after a home delivery with complications were the most common complications. About a third of the women (10 990; 34.9%) had eclampsia or pre-eclampsia and 4856 (15.4%) had an uncomplicated pregnancy.

Out of the 31 501 women admitted, 29 421 (93.4%) indicated they had had at least one antenatal care visit. Of the women with a complicated pregnancy, 89.0% (13 933/15 655) attended at least one antenatal care session, and of the women with (pre-)eclampsia, 97.3% (10 698/10 990) had at least one antenatal care visit.

### Descriptive analysis by outcome

Adverse outcomes by pregnancy category are shown in Table 1. The average number of admissions per month was 588 (median = 578, standard deviation = 94). Of all pregnancies, 204 (0.6%) maternal deaths occurred: 1.1% (119/10 990) of women with (pre)-eclampsia and 0.5% (85/15 655) of women with a complicated pregnancy died. A total of 1962 deliveries (7.9%) resulted in at least one stillbirth: 9.4% (929/9841) of women with a complicated pregnancy had a stillbirth and 10.0% (1033/10 295) of women with (pre-)eclampsia had a stillbirth. For LBW, 11 008/24938 (44.1%) women delivered at least one neonate with LBW: 58.6% of women with (pre-)eclampsia and 50.8% of women with a complicated pregnancy had LBW neonates. None of the uncomplicated pregnancies was associated with maternal death, stillbirth or LBW. Women with (pre-)eclampsia were significantly more likely to die and to have a neonate with LBW than women with a complicated pregnancy (*p* < 0.001). There was no significant difference in the proportion of stillbirths between women with a complicated pregnancy and women with (pre-)eclampsia (*p* = 0.16).

### Regression analysis

**Maternal death.** Caesarean section was a risk factor for maternal death in women with a complicated pregnancy and in women with (pre-)eclampsia (Table 2). After including an interaction term for type of delivery and pregnancy category, the model showed that women with a complicated pregnancy were 4.81 times more likely of dying when having a caesarean section compared to a normal delivery (OR 4.81; 95% CI 1.67–13.84). Women with (pre-)eclampsia were 2.30 times more likely of dying when having a caesarean section compared to a normal delivery (OR 2.30; 95% CI 1.46–3.61). Women with (pre-)eclampsia were 5.62 times more likely to die than women with a complicated pregnancy (OR 5.62; 95% CI 1.97–16.06).

**TABLE 1.** Adverse maternal and neonatal outcomes by pregnancy category for all maternal admissions to Centre de Référence pour les Urgences Obstétricales, Haiti, 2013–2018

Outcome	All pregnancies		Uncomplicated pregnancy		Complicated pregnancy		(Pre-)eclampsia		*p* ^a^
	n	%	n	%	n	%	n	%	
Maternal death									
Yes	204	0.6	0	0.0	85	0.5	119	1.1	< 0.001
No	31 297	99.4	4 856	100.0	15 570	99.5	10 871	98.9	
Total	31 501	100.0	4 856	100.0	15 655	100.0	10 990	100.0	
Stillbirth									
Yes	1 962	7.9	0	0.0	929	9.4	1 033	10.0	0.16
No	23 016	92.1	4 842	100.0	8 912	90.6	9 262	90.0	
Total	24 978	100.0	4 842	100.0	9 841	100.0	10 295	100.0	
Low birthweight									
Yes	11 008	44.1	0	0.0	4 992	50.8	6 016	58.5	< 0.001
No	13 930	55.9	4 838	100.0	4 833	49.2	4 259	41.5	
Total	24 938	100.0	4 838	100.0	9 825	100.0	10 275	100.0	

a The chi-squared test was used to evaluate differences between complicated pregnancy/delivery and (pre-)eclampsia.

**Note**: Uncomplicated pregnancies were defined as a singleton pregnancy without maternal morbidities, low birthweight or delivery complications. A pregnancy with (pre)-eclampsia was defined as one where a woman was admitted between 20 weeks of pregnancy and early postpartum who experienced convulsive seizures in the context of pre-eclampsia without other cause. A complicated pregnancy was defined as all complicated pregnancies without eclampsia and pre-eclampsia, with or without delivery complications.

***Source*:** prepared by authors with the results of the study.

**Stillbirth.** In the multivariable analysis, women older than 35 years were more likely to have a stillbirth in complicated pregnancies and/or deliveries (OR 1.31; 95% CI 1.12–1.55) and (pre-)eclampsia (OR 1.43; 95% CI 1.24–1.65) than women between 20 and 34 years (Table 3). Women younger than 20 years were less likely to have a stillbirth when they had (pre-)eclampsia (OR 0.74; 95% CI 0.57–0.97) compared with women between 20 and 34 years.

No significant association between stillbirth and multiple pregnancy was identified. Not attending antenatal care was a risk factor for stillbirth in women with complicated pregnancies (OR 4.82; 95% CI 3.55–6.55). For all pregnancies, the multivariable model shows that women with (pre-)eclampsia had a 1.34 times higher risk of having a stillbirth than women with a complicated pregnancy (OR 1.34; 95% CI 1.18–1.52) (Table 3).

Women who had a caesarean section had a lower chance of a stillbirth in both pregnancy categories than women who had a normal vaginal or complicated non-instrumental delivery. Women with a complicated pregnancy who had a caesarean section had a 1.41 times lower chance of a stillbirth (OR 0.71; 95% CI 0.62–0.83) compared to such women who had a normal delivery. Women with (pre-)eclampsia had a 3.45 times lower chance of a stillbirth when they had a caesarean section compared to a normal vaginal delivery (OR 0.29; 95% CI 0.24–0.33.

Women with a complicated pregnancy had a 1.69 higher risk of a stillbirth when they had a complicated non-instrumental delivery compared to a caesarean section (OR 0.59; 95% CI 0.43–0.81). In addition, women with (pre-)eclampsia were 2.44 times more likely to have a stillbirth when they had a complicated non-instrumental delivery compared to a caesarean section (OR 0.41; 95% CI 0.20–0.86).

**LBW.** Maternal age was significantly associated with having a LBW baby in women with (pre-)eclampsia (Table 4). Women who experienced eclampsia or pre-eclampsia who were younger than 20 years had a significantly lower risk of having a LBW baby (OR 0.73; 95% CI 0.63–0.83) than women with (pre-)eclampsia aged 20–34 years. Multiple pregnancy was a risk factor for LBW in Women with a complicated pregnancy and women with (pre-)eclampsia had an over five times higher risk of a LBW baby if they had a multiple pregnancy than a singleton pregnancy. Not attending antenatal care was also a risk factor for LBW in women with a complicated pregnancy (OR 1.40; 95% CI 1.05–1.86) (Table 4).

## DISCUSSION

This study analysed 5 years of maternal admission data in women admitted to the Centre de Référence pour les Urgences Obstétricales between 2013 and 2018. Rates of (pre)-eclampsia in the cohort were high with 34.9% of the women experiencing this condition. This finding is not unexpected given that the hospital was set up to manage complicated pregnancies and deliveries, but this rate is more than double the rates of (pre)-eclampsia found in a hospital in rural Haiti (28). This finding indicates inadequate monitoring and detection of complications during pregnancy. This is of concern because our study showed that women with (pre-)eclampsia had the highest risk of maternal death, stillbirth and LBW babies, which concurs with a 2019 study in Haiti (29).

The estimated maternal mortality at the Centre de Référence pour les Urgences Obstétricales over the study period was with 648 women per 100 000 giving birth, which is higher than national maternal mortality rate (529 per 100 000 giving birth) (30). However, this difference is understandable considering the women served by the hospital and the strict admission criteria on high-risk pregnancies. No reliable maternal mortality data in obstetric hospitals outside Port-au-Prince are available, but with less accessibility to health care, it is likely that these rates are even higher in hospitals in rural areas.

**TABLE 2.** Risk factors for maternal death by pregnancy category for all maternal admissions to Centre de Référence pour les Urgences Obstétricales, Haiti, 2013–2018: logistic regression analysis

**Risk factor**	**Complicated pregnancy**					**(Pre-)eclampsia**					**All pregnancies**	
	**Maternal deaths, n (%)** ^a^	**Univariable analysis**		**Multivariable analysis**		**Maternal deaths, n (%)**	**Univariable analysis**		**Multivariable analysis**		**Multivariable analysis**	
		**OR (95% CI)**	***p***	**OR (95% CI)**	***p***		**OR (95% CI)**	***p***	**OR (95% CI)**	***p***	**OR (95% CI)**	***p***
Age group, in years	**85 (0.5)**		0.14			**119 (1.1)**		0.24				
< 20	4 (0.3)	0.52 (0.19–1.43)		NR		11 (1.0)	0.87 (0.46–1.63)	0.65	NR		NA	
20–34	58 (0.5)	reference		reference		87 (1.2)	reference		reference		reference	
≥ 35	23 (0.7)	1.34 (0.83–2.18)		NR		21 (0.8)	0.67 (0.42–1.09)	0.11	NR		NR	
Type of pregnancy	**30 (0.3)**		0.59			**88 (0.9)**		0.047		0.07		
Singleton	28 (0.3)	reference		reference		81 (0.8)	reference		reference		reference	
Multiple	2 (0.4)	1.49 (0.35–6.26)		NR		7 (1.8)	2.20 (1.01–4.79)		2.08 (0.95–4.56)		NR	
Type of delivery	**30 (0.3)**		0.003		0.003	**88 (0.9)**		< 0.001		< 0.001		< 0.001
Normal vaginal	4 (0.1)	reference		reference		28 (0.5)	reference		reference		reference	
Complicated non-instrumental	1 (0.3)	2.73 (0.30–24.46)		2.73 (0.30–24.46)		0 (0.0)	No maternal deaths		No maternal deaths		0.51 (0.07–3.74)	
Instrumental vaginal	0 (0.0)	No maternal deaths		No maternal deaths		0 (0.0)	No maternal deaths		No maternal deaths		NR	
Caesarean section	25 (0.5)	4.78 (1.66–13.76)		4.78 (1.66–13.76)		60 (1.2)	2.36 (1.51–3.71)		2.36 (1.51–3.71)		2.45 (1.63–3.69)	
Antenatal care	**85 (0.5)**		0.42			**119 (1.1)**		0.11				0.64
Yes	78 (0.6)	reference		reference		113 (1.1)	reference		reference		reference	
No	7 (0.4)	0.73 (0.33–1.58)		NR		6 (2.1)	1.97 (0.86–4.50)		NR		0.71 (0.18–2.90)	
Pregnancy type												0.001
Complicated pregnancy											reference	
Eclampsia/pre-eclampsia											5.62 (1.97–16.06)	
Type of delivery # pregnancy type												**< 0.001**
Caesarean section vs normal delivery # complicated pregnancy											4.81 (1.67–13.84)	0.004
Caesarean section vs normal delivery # eclampsia											2.30 (1.46–3.61)	< 0.001

OR, odds ratio; CI, confidence interval, NR, not retained in the final multivariable model, #, interaction term.

a Percentages are calculated from the total number of women in the risk factor category.

**Note**: A pregnancy with (pre)-eclampsia was defined as one where a woman was admitted between 20 weeks of pregnancy and early postpartum who experienced convulsive seizures in the context of pre-eclampsia without other cause. A complicated pregnancy was defined as all complicated pregnancies without eclampsia and pre-eclampsia, with or without delivery complications.

***Source*:** prepared by authors with the results of the study.

**TABLE 3.** Risk factors for stillbirth by pregnancy category for all maternal admissions to Centre de Référence pour les Urgences Obstétricales, Haiti, 2013–2018: logistic regression analysis

**Risk factor**	**Complicated pregnancy**					**(Pre-)eclampsia**					**All pregnancies**	
	**Stillbirths, n (%)** ^a^	**Univariable analysis**		**Multivariable analysis**		**Stillbirths, n (%)**	**Univariable analysis**		**Multivariable analysis**		**Multivariable analysis**	
	**929 (9.4)**	**OR (95% CI)**	***p***	**OR (95% CI)**	***p***	**1 033 (10.0)**	**OR (95% CI)**	***p***	**OR (95% CI)**	***p***	**OR (95% CI)**	***p***
Age group, in years			< 0.001		< 0.001			< 0.001		< 0.001		< 0.001
< 20	71 (7.5)	0.81 (0.63–1.05)		0.78 (0.60–1.01)		67 (6.7)	0.70 (0.54–0.91)		0.74 (0.57–0.97)		0.73 (0.60–0.88)	
20–34	628 (9.1)	reference		reference		640 (9.3)	reference		reference		reference	
≥ 35	230 (11.6)	1.31 (1.12–1.54)		1.31 (1.12–1.55)		326 (13.4)	1.50 (1.30–1.73)		1.43 (1.24–1.65)		1.38 (1.24–1.54)	
Type of pregnancy			0.7					0.2				
Singleton	884 (9.4)	reference		reference		1001 (10.1)	reference		reference		reference	
Multiple	45 (10.0)	1.06 (0.78–1.46)		NR		32 (8.1)	0.79 (0.55–1.14)		NR		NR	
Type of delivery			< 0.001		< 0.001			< 0.001		< 0.001		< 0.001
Normal vaginal	444 (10.9)	reference		reference		759 (14.4)	reference		reference		reference	
Complicated non-instrumental	49 (13.1)	1.23 (0.90–1.69)		1.20 (0.87–1.65)		52 (26.5)	2.14 (1.55–2.97)		2.11 (1.52–2.93)		1.43 (1.15–1.80)	
Instrumental vaginal	1 (3.6)	0.30 (0.04–2.24)		0.30 (0.04–2.21)		4 (12.1)	0.82 (0.29–2.33)		0.80 (0.28–2.28)		0.60 (0.24–1.50)	
Caesarean section	435 (8.1)	0.72 (0.63–0.83)		0.71 (0.62–0.83)		218 (4.5)	0.28 (0.24–0.33)		0.29 (0.25–0.34)		0.47 (0.42–0.51)	
Antenatal care			< 0.001		< 0.001			0.25				< 0.001
Yes	865 (9.1)	reference		reference		1003 (10.0)	reference		reference		reference	
No	64 (31.8)	4.74 (3.49–6.43)		4.82 (3.55–6.55)		30 (12.2)	1.25 (0.85–1.85)		NR		2.64 (2.08–3.34)	
Pregnancy type												< 0.001
Complicated pregnancy											reference	
Eclampsia/pre-eclampsia											1.34 (1.18–1.52)	
Type of delivery # pregnancy type												< 0.001
C-section vs non-instrumental delivery # complicated pregnancy											0.59 (0.43–0.81)	0.001
C-section vs normal delivery # complicated pregnancy											0.71 (0.62–0.82)	< 0.001
C-section vs non-instrumental delivery # eclampsia											0.41 (0.20–0.86)	0.02
C-section vs normal delivery # eclampsia											0.29 (0.24–0.33)	< 0.001

OR, odds ratio; CI, confidence interval, NR, not retained in the final multivariable model, #, interaction term.

a Percentages are calculated from the total number of women in the risk factor category.

**Note**: A pregnancy with (pre)-eclampsia was defined as one where a woman was admitted between 20 weeks of pregnancy and early postpartum who experienced convulsive seizures in the context of pre-eclampsia without other cause. A complicated pregnancy was defined as all complicated pregnancies without eclampsia and pre-eclampsia, with or without delivery complications.

***Source*:** prepared by authors with the results of the study.

**TABLE 4.** Risk factors for low neonatal birthweight by pregnancy category for all maternal admissions to Centre de Référence pour les Urgences Obstétricales, Haiti, 2013–2018: logistic regression analysis

**Risk factor**	**Complicated pregnancy**					**(Pre-)eclampsia**				
	**Low birthweight, n (%)**	**Univariable analysis**		**Multivariable analysis**		**Low birthweight, n (%)**	**Univariable analysis**		**Multivariable analysis**	
	**4 992 (50.8)**	**OR (95% CI)**	***p***	**OR (95% CI)**	***p***	**6 016 (58.6)**	**OR (95% CI)**	***p***	**OR (95% CI)**	***p***
Age group, in years			0.07		0.07			< 0.001		< 0.001
< 20	512 (54.2)	1.17 (1.02–1.34)		1.22 (1.06–1.40)		504 (50.7)	0.71 (0.62–0.81)		0.73 (0.63–0.83)	
20–34	3 472 (50.3)	reference		reference		4 053 (59.2%)	reference		reference	
≥ 35	1 008 (50.9)	1.02 (0.92–1.13)		1.04 (0.94–1.15)		1459 (60.0)	1.04 (0.94–1.14)		1.04 (0.95–1.14)	
Type of pregnancy			< 0.001		< 0.001			< 0.001		< 0.001
Singleton	4 617 (49.3)	reference		reference		5 668 (57.4%)	reference		reference	
Multiple	375 (83.0)	5.02 (3.91–6.43)		5.09 (3.97–6.53)		348 (99.6)	5.75 (4.20–7.86)		5.68 (4.15–7.77)	
Antenatal care			0.03		0.02			0.47		
Yes	4 876 (50.7)	reference		reference		5 867 (58.5%)	reference		reference	
No	116 (58.8)	1.36 (1.02–1.81)		1.40 (1.05–1.86)		149 (60.8)	1.10 (0.85–1.43)		NR	

OR, odds ratio; CI, confidence interval, NR, not retained in the final multivariable model.

**Note**: A pregnancy with (pre)-eclampsia was defined as one where a woman was admitted between 20 weeks of pregnancy and early postpartum who experienced convulsive seizures in the context of pre-eclampsia without other cause. A complicated pregnancy was defined as all complicated pregnancies without eclampsia and pre-eclampsia, with or without delivery complications. Percentages are calculated from the total number of women in the risk factor category.

***Source*:** prepared by authors with the results of the study.

One of the findings of this study is the association between type of delivery and the three negative outcomes. In our study, 40.7% of the women had a caesarean section, mostly women with a complicated pregnancy (54.5%). The risk of stillbirth in neonates of women with a complicated pregnancy or (pre-)eclampsia fell considerably with caesarean section delivery. However, the risk of maternal death was higher in women with a complicated pregnancy or (pre-)eclampsia when they had a caesarean section. That said, considering that the women in the study were at high risk, caesarean sections were more likely to be performed in serious cases that already had a higher risk of dying, which would explain the increased maternal mortality.

Women older than 35 years were more likely to have a stillbirth regardless of their pregnancy category. This association has also been shown in a systematic review on maternal age and the risk of stillbirth (22). Multiple studies have demonstrated that caesarean sections could reduce the number of stillbirths (20, 31) and the results in our study support this finding. For women with a complicated pregnancy or (pre-)eclampsia, a complicated non-instrumental delivery was associated with the highest risk on stillbirth. However, a substantial proportion of these stillbirths delivered by obstetric manipulation are considered preventable as early detection of complications and access to good-quality and timely obstetric care and antenatal care services can facilitate timely action (32).

Of all the women in our study, 93.4% indicated that they had had at least one antenatal care visit. This proportion was significantly lower in women with a complicated pregnancy (89.0%), indicating that some potentially complicated pregnancies and deliveries could be prevented if women attended antenatal care services. Not attending antenatal care services was a risk factor for a stillbirth or having a LBW baby for women with a complicated pregnancy. Attending antenatal care was associated with a lower risk of adverse neonatal outcomes including LBW and stillbirth in women with a complicated pregnancy. Pre-eclampsia can be detected and appropriately managed before the onset of eclampsia and although the only way to end pre-eclampsia is delivery, antenatal care could be an opportunity for the provision of aspirin, magnesium and calcium supplementation (20, 33).

Our results highlight the importance of pregnant women using antenatal care services. However, numerous barriers prevent Haitian women from attending antenatal care, including financial and geographical barriers, lack of service coverage, lack of transportation and poor quality of care (18, 19). For example, a study shows that Haiti only has 0.3 dispensaries per 10 000 inhabitants, which is well below the target of the Ministry of Health and Population in Haiti (16). Measures are therefore needed to improve access to antenatal care in Haiti such as: 1) allocation of more resources to provide free and geographically distributed primary health care; 2) provision of standard antenatal care in all primary health care facilities; and 3) improvement of the quality of antenatal care by providing regular training for health providers.

Since this is a retrospective study using routinely collected data, we faced some challenges. Many of the routinely collected clinical data were not detailed enough to be able to categorize maternal admissions more precisely. No data were available on potentially important confounders such as maternal lifestyle, nutritional status, co-morbidities, socioeconomic status and reproductive health history. In addition, it was not possible to distinguish whether negative outcomes were a (likely) result of a complicated pregnancy or a complicated delivery, or both. This lack of specificity in our dataset therefore also limits the use of the results to guide improvements in the quality of care in obstetric facilities within Haiti in a more targeted manner. Another limitation of this study is that follow-up of the women after discharge from hospital was not possible. Therefore, maternal mortality rates in this study might be an underestimation. It should also be noted that our results are not generalizable across Haiti, because the Centre de Référence pour les Urgences Obstétricales targeted mainly high-risk pregnancies and complicated deliveries in metropolitan Port-au-Prince.

## Conclusions

The study suggests that the prevalence of maternal mortality, stillbirth and LBW remains high in Haiti. In addition, the proportion of women with (pre-)eclampsia was alarmingly high in this obstetric hospital in Port-au-Prince. Although the hospital mainly served women with high-risk pregnancies, it is likely that the prevalence of (pre-)eclampsia and other complications is also high in other areas of Haiti and prevention and treatment are key to reduce maternal death, stillbirth and LBW. Attendance of antenatal care services was an important factor associated with a lower risk of adverse neonatal outcomes including LBW and stillbirth in women with a complicated pregnancy. To prevent and treat pregnancy complications as early as possible, attending high-quality antenatal care services is crucial, especially with high rates of (pre-)eclampsia and other complications as in Haiti. Therefore, improving the quality of and access to antenatal care services and providing it free to all pregnant women in Haiti is recommended.

## Disclaimer.

The authors are solely responsible for the views expressed in the manuscript, which may not necessarily reflect the opinion or policy of the *Revista Panamericana de Salud Pública / Pan American Journal of Public Health* and/or those of the Pan American Health Organization
